# The effects of strategy training on spatial memory in diencephalic amnesia: a randomized controlled study

**DOI:** 10.1007/s10339-020-00961-z

**Published:** 2020-02-17

**Authors:** Roy P. C. Kessels, Sjoerd Murk, Serge J. W. Walvoort, Benjamin M. Hampstead

**Affiliations:** 1 Neuropsychology and Rehabilitation Psychology, Donders Institute for Brain, Cognition and Behaviour, Radboud University, Montessorilaan 3, 6525 HR Nijmegen, The Netherlands; 2 Centre of Excellence for Korsakoff and Alcohol-Related Cognitive Disorders, Vincent van Gogh Institute for Psychiatry, Venray, The Netherlands; 3 Department of Medical Psychology, Radboud University Medical Center, Nijmegen, The Netherlands; 4 Department of Psychiatry, University of Michigan, Ann Arbor, MI USA; 5 VA Ann Arbor Healthcare System, Ann Arbor, MI USA

**Keywords:** Spatial memory, Amnesia, Mnemonic strategies, Neuropsychology, Korsakoff’s syndrome

## Abstract

Alcoholic Korsakoff’s syndrome is characterized by severe amnesia, also affecting spatial memory. To date, research on cognitive rehabilitation in these patients is scarce. Aim of the present study is to examine the efficacy of a mnemonic strategy training in patients with Korsakoff’s syndrome. A randomized controlled exploratory study was performed. A convenience sample of 14 patients with amnesia due to alcoholic Korsakoff’s syndrome was included and randomized into a mnemonic strategy training group (*n* = 7) and a control group (*n* = 7). The training group completed a 3-day 45–60 min mnemonic strategy training that focused on specific strategies to encode and retrieve information about specific objects and their locations in virtual rooms, using labeling, verbal reasoning and mental imagery. The control group only received care as usual. Outcome measure was an object-location memory task consisting of novel, untrained object locations administered 1 day before the intervention, as well as 1 day and 1 week after completing the intervention. Patients in the intervention group were able to acquire and use the strategies, but no significant differences were found between the intervention group and the control group, and no significant change in performance was demonstrated compared to baseline 1 day and 1 week after the intervention. To conclude, the mnemonic strategy training in KS patients did not result in a better spatial memory performance 1 day or 1 week after training completion compared to participation in the regular non-cognitive treatment program that focused on occupational therapy, music therapy and exercise.

## Introduction

Korsakoff’s syndrome (KS) is characterized by profound amnesia for contextual, episodic information, due to bilateral damage to the diencephalon, often in the context of chronic alcohol use disorder (AUD) (Arts et al. 2017). Despite the severe contextual memory deficits, affecting memory for object locations (Kessels et al. 2000), KS patients are under some circumstances still able to acquire new spatial information (Kessels and Kopelman 2012). For example, KS patients were severely impaired at the conscious recollection of object locations in virtual rooms, but had spared implicit knowledge of these object locations even after a 1-week delay (Postma et al. 2008).

Findings like this may also have clinical implications, as spared cognitive functions can be used to overcome cognitive deficits. So far, little research has been done on the effects of mnemonic strategy training in KS (Oudman et al. 2015; Goldstein and Malec 1989), with only one study on spatial memory rehabilitation (Kessels et al. 2007). However, an effective mnemonic strategy training has been developed in the field of older adults with mild cognitive impairment (MCI), often due to Alzheimer’s disease (Hampstead et al. 2012a). In this intervention, patients were trained to use specific mnemonic strategies to encode and retrieve information about specific objects and their locations in a virtual room. Participants were instructed to use feature identification (*labeling* a salient feature near the targeted object), verbal reasoning linking the feature to that object (*why* is the object placed there) and mental imagery (making a *mental picture* of the object and its feature). Positive results were demonstrated (Hampstead et al. 2012a), with some evidence that the training resulted in restoration of hippocampal activity in MCI (Hampstead et al. 2012b). The current study investigates the efficacy of this mnemonic strategy training in amnesic patients with KS.

## Methods

### Participants

Fourteen patients with alcoholic KS participated in this study. All were inpatients of the Centre of Excellence for Korsakoff and Alcohol-Related Cognitive Disorders in Venray, the Netherlands. All patients fulfilled the DSM-5 (2013) criteria for Alcohol-Induced Major Neurocognitive Disorder, Confabulatory/Amnestic Type and the criteria for Korsakoff’s syndrome (Arts et al. 2017). The patients had a history of AUD with nutritional depletion, and their diagnoses were substantiated by extensive neuropsychological assessment, psychiatric and neurological examination, and neuroradiological findings. None of the patients fulfilled the criteria for alcohol-related dementia; all were in the chronic stage of the Wernicke–Korsakoff Syndrome, and at least 3 months abstinent from alcohol. Seven patients (5 men; mean age 58.9, SD = 10.9; mean years of education 9.9, SD = 2.5) were at random allocated to the mnemonic strategy training (MST) group and 7 to treatment-as-usual (TAU) group (4 men; mean age 62.6, SD 4.8; mean years of education^1^ 10.3, SD = 2.8). No significant differences on any of the demographic variables were found between both groups (all *t*-values ≤ 0.823). The study design was approved by the Institutional Review Board of Vincent van Gogh Institute of Psychiatry, and written informed consent was obtained from all patients.

### Outcome measure and analyses

All participants completed a computerized object-location task (Hampstead et al. 2012a) 1 day before the start of the treatment or TAU period (baseline), 1 day post-treatment and 1 week after treatment completion (follow-up), with parallel versions being used for each assessment. In this task, the location of 24 everyday, easy-to-name objects in 6 different rooms had to be remembered. In each trial in the learning phase, an object was shown in the middle of the computer screen, after which it was shown at a specific location in one of the rooms for 10 s with the instruction to memorize its location. After 24 learning trials, the participant had to indicate each object’s correct location from five options, presented in a different order than the learning phase. No further instructions on strategy use were provided during either the learning phase or the test phase, other than procedural clarification. For each assessment, different objects were used, and none of the objects or rooms were used in the memory strategy training.

The number of correct object locations was recorded for each assessment (max. = 24) and analyzed using General Linear Model repeated-measures analysis. Also, we examined the individual performances for the post-treatment and 1-week follow-up assessment using the 20% Change Index, indicating whether an individual improved 20% or more compared to his/her baseline performance (Collie et al. 2002).

### Training

**Fig. 1:**
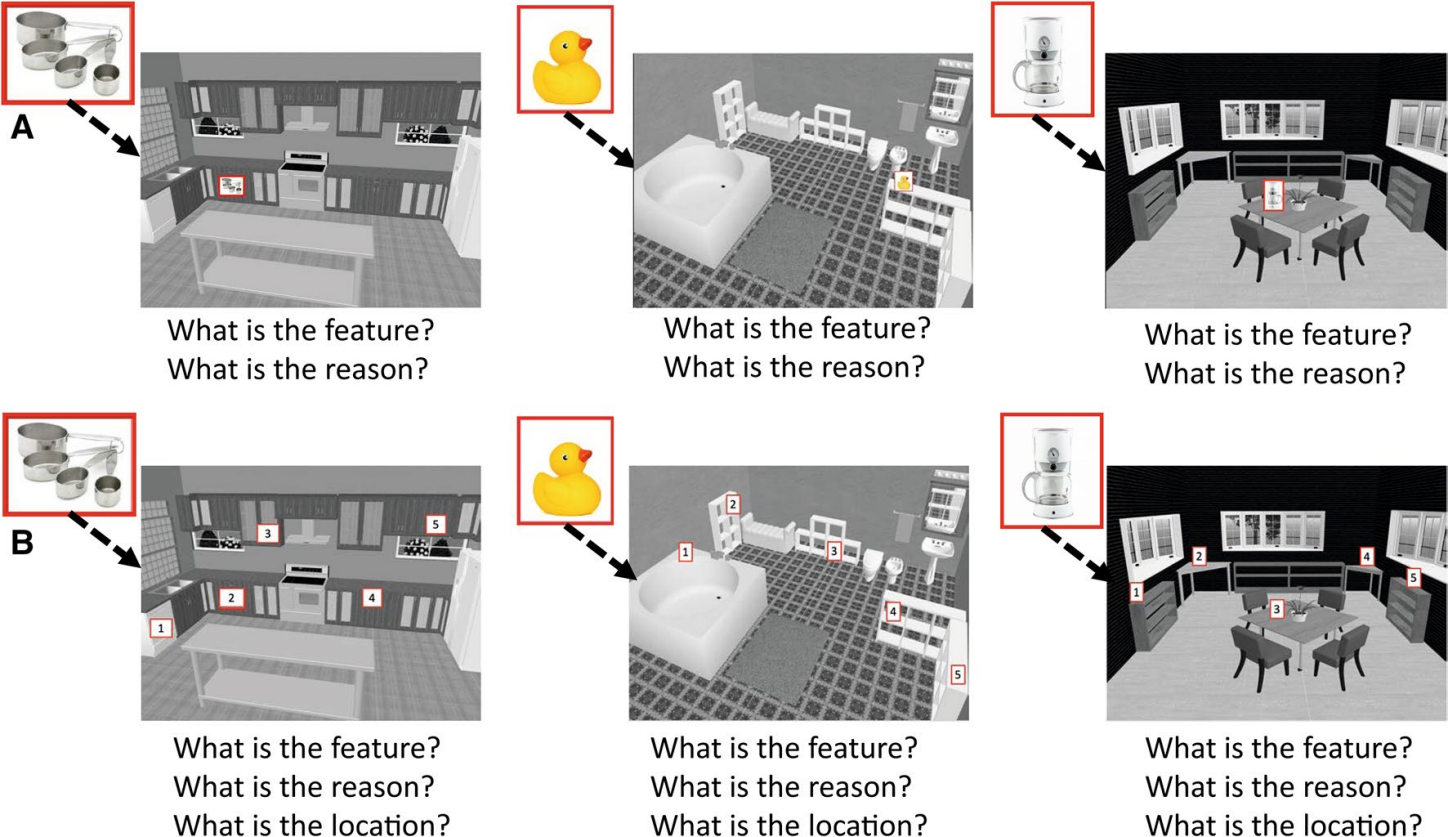
Schematic overview of the object-location memory task that was used to assess the performance pre-treatment, post-treatment and 1 week after treatment, and in the mnemonic strategy training. **a** During the mnemonic strategy training sessions, participants were prompted to use the strategy steps to memorize the object locations (*feature*–*reason*–*image).***b** On subsequent trials, participants were required to actively use the strategies to retrieve the reference point (*feature*), the rationale why the object was placed there (*reason*) and then its *location*, after which feedback was provided

The MST was slightly adapted from the MCI training to make it feasible for use in Korsakoff patients who typically have more severe cognitive deficits. That is, fewer object locations were trained (6 vs. 15 in each session) and fewer repetitions were provided for each stimulus (6 vs. 9). The three training sessions for the MST group took place on consecutive days. In the first, the three steps of the memory strategy were explained, after which the participant was asked to explain the steps to make sure they understood. Steps were presented on paper throughout the memory training as a reminder. Before and after each subsequent session, participants were asked to briefly explain the strategy steps again. Each training session started with a learning session, in which 6 object locations were shown using a computerized task, similar to the object-location memory test (see Fig. 1). Each object was first presented in the middle of the screen, after which the object was shown in the virtual room at a specific location, and prompts about the strategies were presented on the screen. Then the participant had to come up with adequate reference points (*feature*) and a rationale on why the object was placed there (*reason*) with some help from the trainer (Hampstead et al. 2012a, b).

After the learning phase, the actual training phase started, in which the objects were again presented, with 5 possible locations marked in the virtual rooms. Participants were actively prompted to use the strategy steps to retrieve the object locations. Feedback was provided by the trainer about the accuracy of the feature, reason and location. In case of an incorrect response, the feature, reason and location that the participant stated during the learning phase were repeated again. The training phase was repeated 6 times in a different order, so participants had substantial practice with the strategies. The sessions took 45–60 min.

The TAU group did not practice with cognitive strategies during the 3-day period. Both groups participated in the regular treatment program that consisted of music therapy, occupational therapy and movement therapy.

## Results

**Fig. 2:**
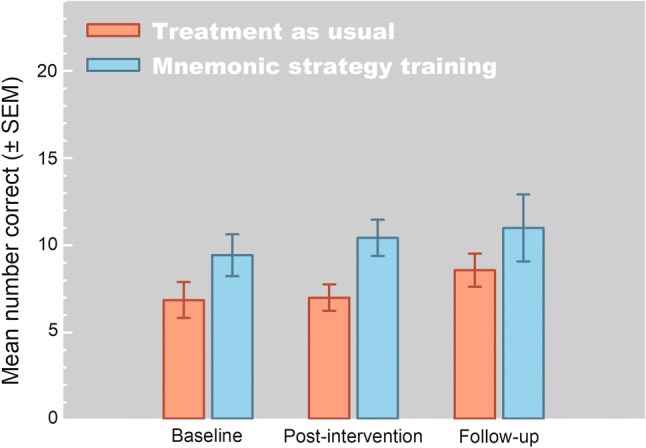
Mean performance (± SEM) on the object-location memory task for the baseline, post-treatment, and 1-week follow-up assessments for the mnemonic training and treatment-as-usual groups

All 14 patients completed all assessments and those in the MST group completed all training sessions and were able to come up with cues themselves in the majority of the trials (66.7%; SD = 13.2). Figure 2 shows the results for the MST and TAU groups. Neither a significant main effect of treatment group [F(1,12) = 3.85, *p* = 0.073, η_p_^2^ = .24], nor a main effect of assessment moment [F(2,24) = 2.17, *p* = 0.136, η_p_^2^ = 0.15] were found, and no time × group interaction [F(2,24) = 0.23, *p* = 0.798, η_p_^2^ = .02]. The between-group effect size for the post-treatment assessment was 0.22 and for the follow-up assessment − 0.15, which are considered small. The distribution of patients who improved more than 20% compared to baseline performance did not differ post-training (MST: *n* = 3, TAU: *n* = 3) or 1 week after training completion [MST: *n* = 3, TAU = 5; *χ*(1) = 1.17, *p* = 0.28].

## Discussion

The feasibility and efficacy of a mnemonic strategy training in KS patients on spatial memory performance were studied. Compared to a control group of KS patients, those who completed the 3-session intervention did not perform better on an object-location memory test of novel materials, in contrast to previous results showing beneficial effects of this training in patients with MCI (Hampstead et al. 2012b). One explanation for this discrepancy may lie in the severity of the amnesia, which may have been more severe in the KS patients than in the MCI sample. Consequently, the strategies that were trained in this intervention may have been too complicated for the patients to independently apply to novel stimuli, despite patients’ ability to learn the steps involved with the strategies. Another explanation is that KS patients also show executive dysfunction (Moerman-Van den Brink et al. 2019), which may have affected the ability of KS patients to benefit from effectively applying mnemonic strategies. Previous research indeed showed that strategies became less effective as the severity of executive impairment increased (Hampstead et al. 2012a). Furthermore, training duration was relatively short (3 sessions over 3 consecutive days) and more intensive intervention, with more learning trials, may be needed in this population.

Remarkably little research has been done on cognitive rehabilitation in patients with alcohol-related brain damage (including KS); with the exception of the study by Yoyman et al. (1988; *n* = 76), all are small-sample group (4–16 patients) or single-case studies (Svanberg and Evans 2013). In non-KS individuals with cognitive impairments due to AUD, evidence for the beneficial effects of memory training is also mixed. That is, a 6-week memory training in 14 AUD patients showed only small gains (Steingass et al. 1994), while Yohman et al. (1988) did not show any significant improvement after a 12-h memory training in 25 AUD patients.

The present study design has several limitations. It is unclear whether mnemonic strategies would facilitate memory for “trained” stimuli, as in earlier trials with MCI (Hampstead et al. 2012a). Additionally, we used untrained objects and rooms as the outcome measure, but did not include a different type of memory test to investigate near-transfer effects. However, the lack of a positive finding on the object-location memory task makes it unlikely that such a near-transfer would have been present in our sample. Another limitation is the small sample size, albeit small effect sizes do not point toward a lack of power. Also we lack a standardized set of neuropsychological measures in our group that can be individually related to training gain, something which should be explored in future research. Our findings, in combination with prior evidence (Hampstead et al. 2012a) and models (Hampstead et al. 2014), highlight the differences between intervention approaches and need to better identify the patient characteristics associated with efficacy for the various cognitive intervention techniques.

## Footnotes

1 Reliable data on educational attainment were lacking in 2 patients.
